# Neural stem/progenitor cell‐derived extracellular vesicles: A novel therapy for neurological diseases and beyond

**DOI:** 10.1002/mco2.214

**Published:** 2023-02-07

**Authors:** Xiangyu Li, Yingbo Zhu, Yi Wang, Xiaohuan Xia, Jialin C. Zheng

**Affiliations:** ^1^ Center for Translational Neurodegeneration and Regenerative Therapy Tongji Hospital, Tongji University School of Medicine Shanghai China; ^2^ Center for Translational Neurodegeneration and Regenerative Therapy Yangzhi Rehabilitation Hospital, Tongji University Shanghai China; ^3^ Shanghai Frontiers Science Center of Nanocatalytic Medicine Tongji University School of Medicine Shanghai China; ^4^ Translational Research Institute of Brain and Brain‐Like Intelligence Shanghai Fourth People's Hospital, Tongji University School of Medicine Shanghai China; ^5^ Key Laboratory of Spine and Spinal Cord Injury Repair and Regeneration, Tongji University Ministry of Education Shanghai China

**Keywords:** exosomes, extracellular vesicle, neural stem/progenitor cell, neuroinflammation, neurological disease, neuroregeneration

## Abstract

As bilayer lipid membrane vesicles secreted by neural stem/progenitor cells (NSCs), NSC‐derived extracellular vesicles (NSC‐EVs) have attracted growing attention for their promising potential to serve as novel therapeutic agents in treatment of neurological diseases due to their unique physicochemical characteristics and biological functions. NSC‐EVs exhibit advantages such as stable physical and chemical properties, low immunogenicity, and high penetration capacity to cross blood–brain barrier to avoid predicaments of the clinical applications of NSCs that include autoimmune responses, ethical/religious concerns, and the problematic logistics of acquiring fetal tissues. More importantly, NSC‐EVs inherit excellent neuroprotective and neuroregenerative potential and immunomodulatory capabilities from parent cells, and display outstanding therapeutic effects on mitigating behavioral alterations and pathological phenotypes of patients or animals with neurological diseases. In this review, we first comprehensively summarize the progress in functional research and application of NSC‐EVs in different neurological diseases, including neurodegenerative diseases, acute neurological diseases, dementia/cognitive dysfunction, and peripheral diseases. Next, we provide our thoughts on current limitations/concerns as well as tremendous potential of NSC‐EVs in clinical applications. Last, we discuss future directions of further investigations on NSC‐EVs and their probable applications in both basic and clinical research.

## Graphical abstract

After administration, NSC‐EVs can be internalized by various brain cells and vascular endothelial cells to exert their therapeutic effects. After being internalized by different types of brain cells, NSC‐EVs promote the regenerative potential of endogenous NSCs, inhibit the excessive activation and neurotrophin production of astrocytes, rescue neuronal and synaptic damage from oxidative stress, promote remyelination capacity of oligodendrocytes, modulate inflammatory responses of resident microglia, and stimulate endothelial cell vascular regeneration, therefore ameliorating neurological disorders and accelerating post‐disease recovery.

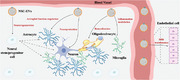

## 1 INTRODUCTION

With the rapid growth of aging population over the past decades, the incidence of neurological diseases, including neurodegenerative diseases and acute neural injury, has increased year by year.^1^, ^2^ These diseases are characterized by pathological features such as the loss of neuronal cells, excessive inflammatory responses of glial cells, impaired neuroregeneration, and disrupted blood–brain barrier (BBB) functions. Numerous clinical trials that focus on eliminating one single pathological feature of neurological diseases end up with unsatisfied outcomes, suggesting the necessity to develop novel therapeutic strategies for these intractable diseases.

Neural stem/progenitor cells (NSCs) possess proliferation and multipotent differentiation capabilities. Over the past few decades, the application of NSCs has emerged as a promising therapeutic strategy in the treatment of various neurological disorders, including chronic neurodegenerative diseases (e.g., Alzheimer's disease [AD], Parkinson's disease [PD], amyotrophic lateral sclerosis [ALS]) and acute central nervous system (CNS) damages (e.g., ischemic stroke [IS], spinal cord injury [SCI]).^3^ Brain‐transplanted NSCs isolated from fetal tissues were reported to differentiate into corresponding neurons that were able to repair damaged neurons.^4^, ^5^, ^6^ In addition, NSCs release neurotrophic factors to restore CNS microenvironment, leading to the alleviation of disease phenotypes.^7^, ^8^ However, NSC application receives great constraints due to safety issues, cellular immunogenicity issues, one‐off effects, ethical and logical problems of acquiring fetal tissues, and tumor risk.^9^, ^10^ In this case, scientists have started to convert their attention from NSCs to NSC‐derived extracellular vesicles (NSC‐EVs) to overcome aforementioned concerns.

EVs, small bilipid layer‐enclosed structures released from most eukaryotic cells, are found in tissues and biological fluids.^11^, ^12^, ^13^ Mounting evidence has shown that NSC‐EVs inherit abundant neuroprotective and neuroregenerative non‐coding RNAs, proteins, lipids, and other active components from their parent cells.^14^, ^15^ The small size and low immunogenicity of NSC‐EVs allow them to avoid being recognized and cleared by the host's immune system after they are administered.^16^, ^17^ EVs’ bilayer membranes and stable physicochemical properties protect EV contents from being degraded in the microenvironment, allowing these contents to function as intercellular communicators.^18^, ^19^ Peripherally administered EVs can move across the BBB by the process of transcytosis, thus delivering their bioactive cargos to the brain.^20^ Moreover, the surface and contents of EVs can be further modified via EVs engineering editing technology to enable EVs to target specific cells and achieve specific biological/therapeutic effects.^21^, ^22^, ^23^ To date, the therapeutic potential of NSC‐EVs has been widely investigated in various neurological disease models.^24^, ^25^, ^26^, ^27^, ^28^ Inspiringly, the application of NSC‐EVs has obtained even greater outcomes than that of mesenchymal stem cell (MSC)‐derived EVs (MSC‐EVs), suggesting that NSC‐EVs have promising therapeutic potential on cell and animal models of neurological diseases.^24^, ^26^, ^29^, ^30^, ^31^

In this review, we comprehensively summarized the biological function and therapeutic effects of NSC‐EVs in treatment of neurological diseases, and discussed the present problems in NSC‐EV application to suggest future directions in this field.

## 2 NSCs: MULTIPOTENT STEM CELLS WITHIN THE CNS

**FIGURE 1:**
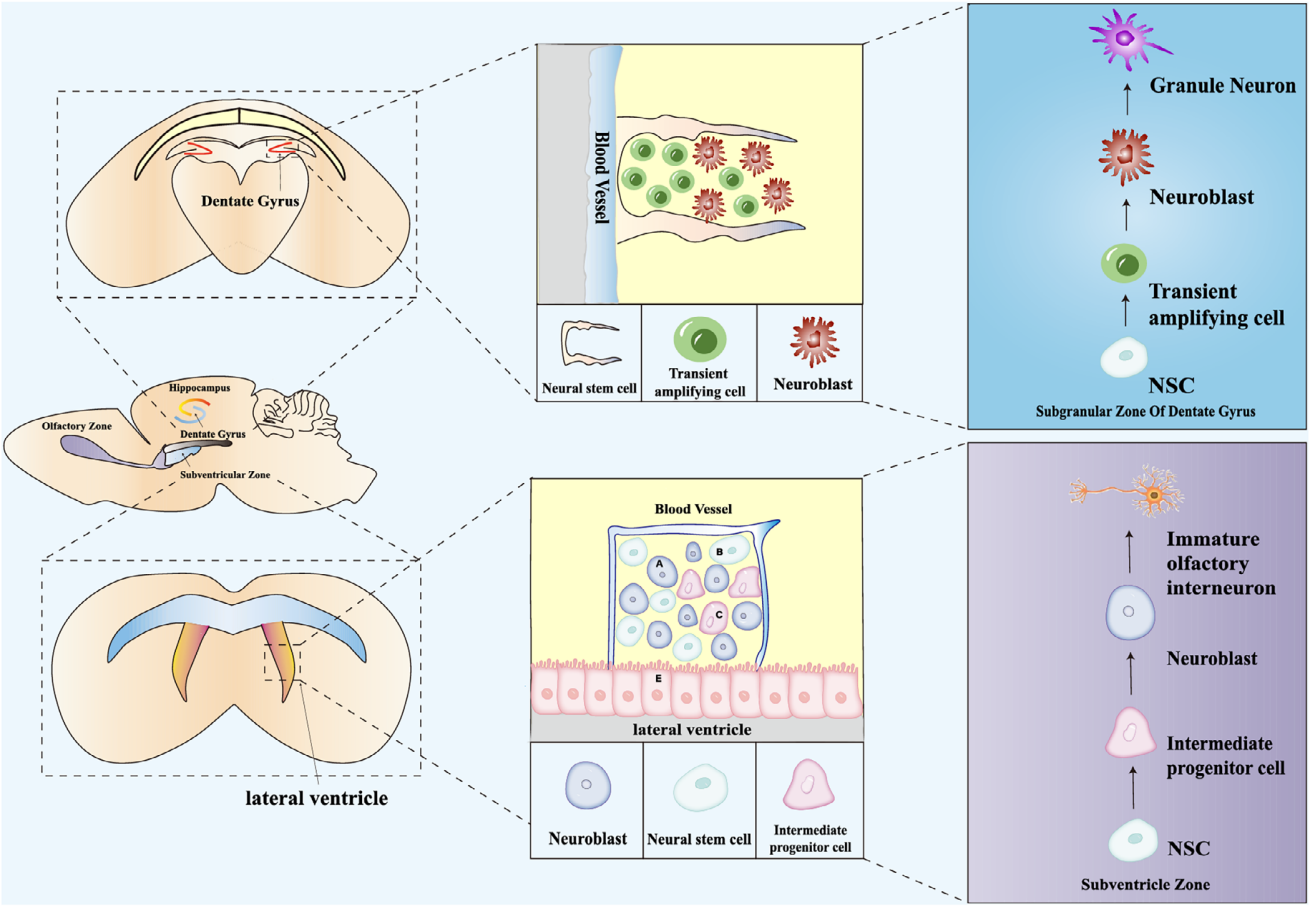
The process of adult neurogenesis. Neurogenic niches in the adult brain are the subventricular zone (SVZ) and the subgranular zone (SGZ). Neural stem cells that are able to self‐renew and differentiate into both neuronal and glial lineages are present in the neurogenic niche. Newborn neurons migrate to different brain regions to exert their functions. SGZ‐derived newborn neurons migrate short distances to be merged into the dentate gyrus (DG) circuits, and SVZ‐derived newborn neurons move far from the niche through the rostral migratory stream (RMS) into the olfactory bulb (OB). SVZ lines the lateral walls of lateral ventricles and is composed of three major cell types: neural stem cells (type B cells) give rise to intermediate progenitor cells (type C cells), which in turn generate neuroblasts (type A cells). After that, neuroblasts differentiate into immature olfactory interneurons and comprise existing neuronal networks. In the SGZ, neural stem cells are activated into nonradial cells and generate transient amplifying cells which in turn give rise to neuroblasts. Neuroblasts convert into immature neurons that migrate to the inner granule cell layer and differentiate into granule neurons.

NSCs are multipotent stem cells that inhabit in the CNS with the capacity to self‐renew and differentiate into both neuronal and glial lineages (Figure 1).^32^ The discovery of NSCs can be traced back to the 1960s. With continuous development of autoradiography, scientists first discovered newborn neurons in the olfactory bulb (OB), subventricular zone (SVZ), hippocampus, spinal cord, cerebral cortex, and cerebellum in young rats, suggesting neurogenesis in the adult brain.^33^ Afterwards, Altman and Das^34^ found that certain adult rat brain regions including the subgranular zone (SGZ), SVZ, and OB also contain NSCs with the potential for proliferation and differentiation.

To date, NSC transplantation‐based therapy has been widely investigated for its feasibility and effectiveness to rescue the pathological changes in neurological diseases using various disease animal models.^18^, ^35^, ^36^, ^37^ NSCs exhibit the capacity to replace degenerating or damaged neurons and secrete neuroprotective and immunomodulatory substances such as brain‐derived neurotrophic factor (BDNF) and glia‐derived neurotrophic factor after transplantation.^38^, ^39^, ^40^ For example, NSC transplantation has been reported to improve the cognitive functions and enhance synaptogenesis in AD models,^41^ attenuate induced turning behavior in PD models,^42^ preserve motor neurons and muscle innervation in ALS models,^43^ and enhance dendrite branching and corticospinal tract projections in stroke mice.^44^

Given promising therapeutic potential of NSCs post‐transplantation in animal studies, various clinical trials have been conducted to evaluate the feasibility of NSC transplantation (Table 1).^45^, ^46^, ^47^ Currently, ClinicalTrial database (www.clinicaltrials.gov) lists around 100 clinical trials that examine the safety, distribution, and therapeutic outcome of NSC transplantation that are either completed, ongoing, or planned. Among them, many trials focus on ALS. Multiple phase I/II clinical trials have suggested that all subjects tolerated NSC spinal cord implantation procedures and have shown no serious adverse events including cord damage, syrinx, or tumor formation over 2 years post‐grafting.^45^, ^46^, ^48^, ^49^, ^50^, ^51^, ^52^, ^53^ NSC transplantation seems to improve the clinical states of ALS patients; however, no significant difference was detected when looking at slopes of disease progression since most aforementioned clinical trials were neither designed nor powered to measure treatment efficacy.^48^ In a case series study, NSC transplantation enhanced dopaminergic activity that could correlate with improved motor function in patients with PD, another neurodegenerative disease.^54^ Besides, NSC transplantation was also performed on patients with other neurological diseases. For instance, a multicenter prospective single‐arm study found that NSC administration via stereotactic ipsilateral putamen injection was safe and well tolerated for chronic IS patients.^55^, ^56^, ^57^ More importantly, patients who received NSC transplantation showed increased deep gray matter functional connectivity and improvements in upper limb function. Moreover, phase II studies found that NSC transplantation enhanced the motor function of patients with traumatic cervical SCI, supported by 12 months of clinical follow‐up, implicating great therapeutic potential of NSC transplantation for treating SCI.^45^, ^58^ Meanwhile, a multicenter study indicated that the outcome of NSC transplantation was below the required clinical efficacy threshold set by the sponsor to support further development and resulted in early study termination.^59^ The controversy of clinical trial results could be either due to the small number of participants in these studies or the differentiated effects of NSC transplantation on diverse neurological diseases. Furthermore, multiple clinical pilot studies have shown the feasibility and promising therapeutic potential of NSC transplantation for neonatal brain injury,^60^ inherited cerebellar atrophy,^61^ and severe cerebral palsy.^62^ Hence, inspiring results of NSC transplantation have been obtained by clinical trials; however, it is urgent to conduct more clinical studies to fully validate the safety and therapeutic potential of NSC transplantation.

**TABLE 1:** Summary of the application of neural stem cells (NSCs) in clinical trials

Disease	Phase of study	NSC type	Administration root	Outcome	Ref.
ALS	Phase I	NSI‐566RSC hNSC line	Intraspinal injection	All subjects tolerated the treatment well without long‐term complications related to NSC injection and transplantation surgery	^53^
	Phases I and II	CNS10 hNSC line with ectopic GDNF	Intraspinal injection	Grafted NSCs survive and produce GDNF	^50^
		Human fetal‐derived NSCs	Intraspinal injection	NSC injection and transplantation surgery show no serious adverse effects	^49^, ^51^
	Phase I	NSI‐566RSC hNSC line	Intraspinal injection	NSC injection and transplantation surgery are feasible and well‐tolerated	^52^
	Phases I and II	Human spinal cord‐derived NSCs	Intraspinal injection	NSC transplantation is safe evaluated for adverse events	^48^
PD	A case series study	Human fetal‐derived NSCs	Stereotactic injection	NSC injection and transplantation surgery are safe without immune responses and adverse effects, and improve dopaminergic neurotransmission correlating with improved motor function	^54^
SCI	Phase I	NSI‐566 hNSC line	Intraspinal injection	Subjects tolerated the procedure well without serious adverse events over 27 months post‐grafting	^46^
	Phases I and II	Human fetal‐derived NSCs	Intraspinal injection	Subject showed increased motor scores, recovery of motor levels, and responses to electrophysiological studies without cord damage, syrinx or tumor formation, neurological deterioration, and exacerbating neuropathic pain or spasticity	^45^
	Phase II	Human fetal‐derived NSCs	Intraspinal injection	NSC transplantation improved overall mean functional outcomes of patients without safety issues	^58^
IS	Phase I	CTX0E03 hNSC line	Stereotactic ipsilateral putamen injection	Intracerebral implantation of NSCs is feasible and improves upper limb function of patients	^55^, ^56^, ^57^
Neonatal brain injury	Clinical pilot study	Human fetal‐derived NSCs	Stereotactic injection	NSC transplantation effectively treat patients with severe cortical visual impairment after neonatal brain injury	^60^
Cerebral palsy	Clinical pilot study	Human fetal forebrain‐derived NSCs	Stereotactic injection	NSC transplantation enhances the development of gross motor, fine motor, and cognition function of patients	^61^
Inherited cerebellar atrophy	Clinical pilot study	Human fetal cerebellum‐derived NSCs	Stereotactic injection	NSC transplantation is a feasible and effective treatment for inherited cerebellar atrophy	^62^

Abbreviations: ALS, amyotrophic lateral sclerosis; GDNF, glia‐derived neurotrophic factor; hNSC, human neural stem cell; IS, ischemic stroke; PD, Parkinson's disease; SCI, spinal cord injury.

Although NSCs display excellent therapeutic potential, there are concerns restricting the clinical application of NSCs. Firstly, given the proliferation and differentiation ability of NSCs, it is difficult to control the fate commitment of NSCs post‐transplantation. In fact, mounting studies have demonstrated insufficient differentiation of transplanted NSCs and unsuccessful integration of transplanted cells with endogenous neurons.^63^, ^64^ Secondly, because major histocompatibility complex I (MHCI) and major histocompatibility complex II (MHCII), which can induce lymphocyte proliferation, are expressed on the surface of NSCs, NSCs are likely to have certain immunogenicity and induce immunological rejection.^65^ Thirdly, in most cases, the effect of NSCs is a “one‐shot” effect, but most neurological diseases need long‐term effects to prevent or delay disease progression.^66^ Lastly, although literatures did not report the risk of tumor formation post‐NSC transplantation,^67^ more investigations are required to validate the biological safety of NSCs in more pre‐clinical and clinical trials.^68^, ^69^

Therefore, although NSCs have obvious advantages in the treatment of neurological diseases, we cannot ignore the concerns of safety and others on the clinical application of NSCs, indicating that there is an urgent need for an alternative approach that retains the therapeutic effect of NSC transplantation while being able to avoid aforementioned issues.

## 3 EVs: BIOGENESIS, COMPOSITION, CHARACTERIZATION, AND UPTAKE

EVs are cell‐derived lipid bilayer vesicles (30–2000 nm) that are classified into exosomes, apoptotic bodies, and ectosomes/microvesicles, including blebbing vesicles, shedding vesicles, oncosomes, and migrasomes.^70^, ^71^ EVs are highly heterogeneous due to the distinct size and biogenesis modes of different types of EVs.^71^ For instance, exosomes are an endosome source of vesicles, ectosomes are derived from plasma membrane budding, and apoptotic bodies are generally remnants/fragments of apoptotic cells.^72^ Therefore, different EVs may carry diverse bioactive cargos and exhibit distinct biological functions. In this review, we focused on exosomes and ectosomes, and excluded apoptotic bodies since NSC‐EVs are collected from living cells.

### 3.1 Biogenesis of EVs

**FIGURE 2:**
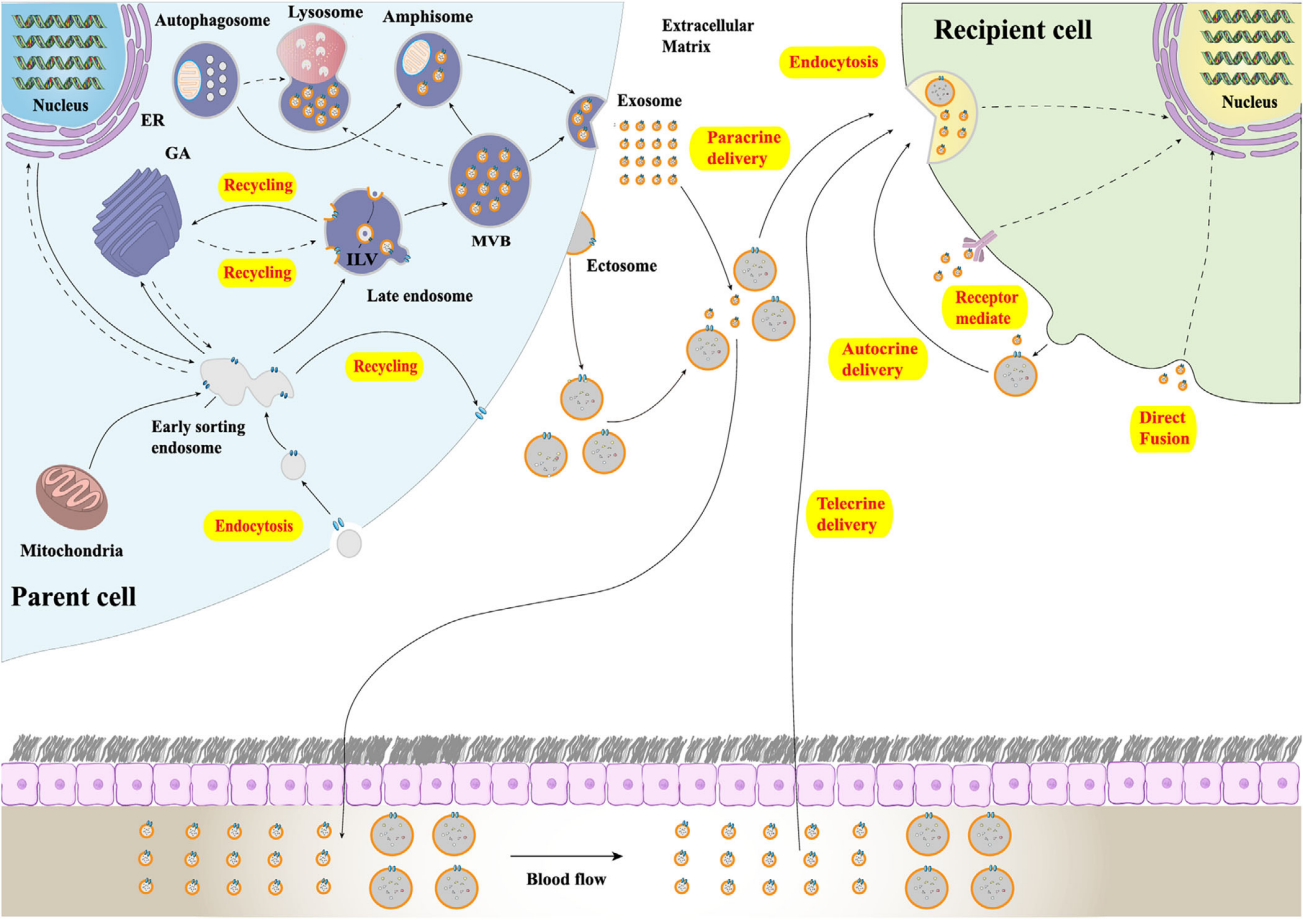
The biogenesis and internalization of extracellular vesicles (EVs). Ectosomes are generated by direct outward budding of cell membrane. Exosome biogenesis initiates from the inward budding of plasma membrane to early sorting endosomes (ESEs) that can communicate with intracellular organelles, be recycled into cell surfaces, or mature into late endosomes (LEs)/multiple vesicular bodies (MVBs). MVBs can either merge into autophagosomes/lysosomes for degradation or be transported to the plasma membrane to release intraluminal vesicles (ILVs) into the extracellular space to form exosomes. In the extracellular space, EVs reach their recipient cells through paracrine, telecrine, and autocrine deliveries. EVs bind to the surface of recipient cells to activate the surface receptors and induce intracellular signaling pathways. EVs can also fuse with recipient cells directly or be internalized via endocytosis, resulting in the release of bioactive cargos from EVs into recipient cells.

To date, distinct biogenesis pathways of ectosomes and exosomes have been discovered (Figure 2).^72^ Ectosomes originated from direct plasma membrane budding. The release of ectosomes requires their separation from the plasma membrane. This mechanism mainly depends on the interaction between actin and myosin and adenosine triphosphate (ATP)‐mediated energy supply.^73^, ^74^ Recent studies have shown that the activation of small guanosine triphosphate binding proteins such as ARF6 and ARF1 leads to the phosphorylation of myosin light chain and the contraction of actin, so that ectosomes are detached from the cell surface.^75^, ^76^ Interestingly, endosomal sorting complex required for transport (ESCRT) protein TSG101 and ATPase VPS4, which are mainly involved in the exosome synthesis pathway, are also involved in the release of ectosomes, suggesting potential crosstalk between the release of ectosomes and exosomes.^77^, ^78^

Unlike that of ectosomes, the formation processes of exosomes, an endosome source of vesicles, are more complex. The biogenesis of exosomes starts from the formation of intraluminal vesicles (ILVs) in late endosomes/multivesicular bodies (MVBs), in which ESCRTs serve as main players. ESCRT complexes (ESCRT‐0, ‐I, ‐II, and ‐III) bind to MVB membrane to regulate the entry and formation of ILV^79^; ESCRT‐0 recognizes monoubiquitinated proteins through hepatocyte growth factor‐regulated tyrosine kinase substrate heterodimers and STAM1/2.^80^, ^81^ ESCRT‐I and ESCRT‐II form a strong recognition domain with high affinity for ubiquitinated substrate of the endosomal membrane and mediate the final budding process.^82^ Finally, ESCRT‐III converges with the complex to cleave the membrane and release buds into the endosome.^83^ Afterwards, MVBs either fuse with lysosomes to be degraded or fuse with the plasma membrane to release ILVs into the extracellular environment to form exosomes.^84^, ^85^, ^86^ Afterwards, exosomes are released into extracellular spaces. For exosome release in the CNS, Rab family (e.g., Rab27A and Rab27B), soluble N‐ethyl maleimide‐sensitive factor attachment protein receptor (SNARE) complex, and synaptic binding proteins functioning as calcium sensors are key mediators.^84^

In reality, however, differentiating ectosomes from exosomes based on their biogenesis pathway is extraordinarily difficult unless the particular EV is caught in the act of release through live imaging; otherwise, the nomenclature of “exosomes” or “ectosomes” is biologically misleading.^87^ Under this circumstance, the Minimal Information for Studies of Extracellular Vesicles (MISEV) guidelines of the International Society for Extracellular Vesicles for EV separation/enrichment urges researchers to consider using operational terms for EV subtypes that refer to (1) physical characteristics of EVs, such as size (e.g., small EVs and medium/large EVs with defined ranges, for instance, <200 nm as small EVs and >200 nm as large/medium EVs); (2) biochemical composition of EVs (e.g., CD63^+^/CD81^+^‐EVs, Annexin A5‐stained EVs); or (3) descriptions of donor cells (e.g., NSC‐EVs, neuron‐derived EVs) instead of terms such as “exosomes” and “ectosomes/microvesicles” that are historically loaded with unrealistic expectations of unique biogenesis pathways.^88^ Therefore, we follow consensus nomenclature and use “EVs” in the review unless reliable protocols have been utilized for isolating exosomes or ectosomes.

### 3.2 Composition and characterization of EVs

**FIGURE 3:**
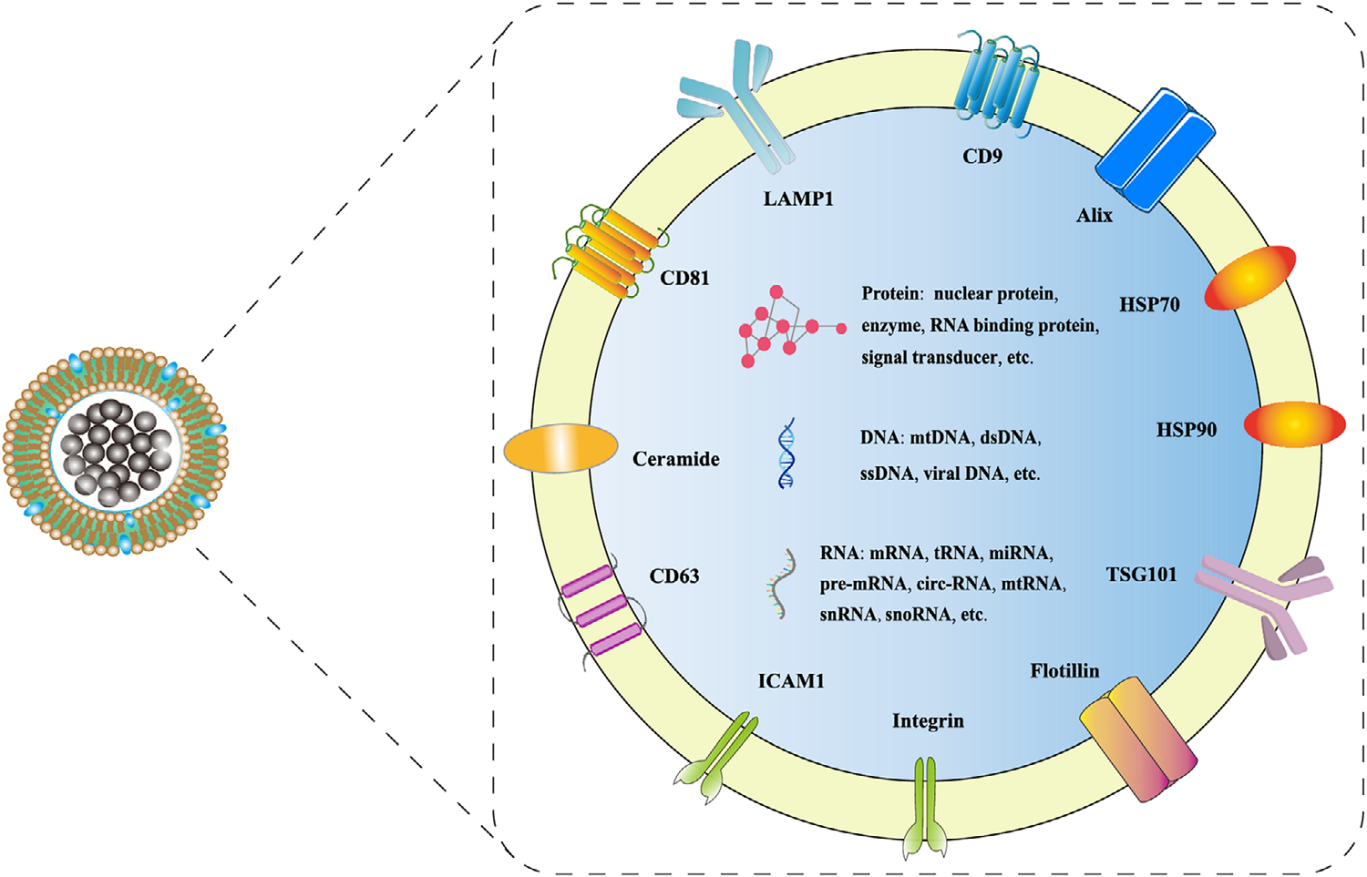
The structure and composition of exosomes. Exosomes are membrane‐surrounded structures released outside the cells. Exosome surface proteins include four transmembrane protein families (e.g., CD9, CD63, CD81), heat shock proteins (e.g., HSP70, HSP90), cytoskeletal protein Alix, intercellular adhesion protein ICAM‐1, integrin, multidomain cytosolic protein TSG101, lipid‐raft‐associated membrane protein Flotillin, lysosome‐associated membrane protein LAMP‐1, ceramide, and more. Exosomes also comprise various intracellular proteins, DNAs, and RNAs (e.g., mRNAs, miRNAs, lncRNAs, etc.) with diverse biological functions.

EVs contain many key nucleic acids and proteins that regulate gene transcription and signal transduction.^72^ To date, more than 9700 proteins, 3400 mRNAs, and 2800 miRNAs have been identified in exosomes (http://www.exocarta.org). Among them, many are membrane proteins expressed on the surface of exosomes, including annexins, flotillin, and tetraspanins (e.g., CD63, CD81, CD9) (Figure 3). These proteins help scientists to characterize and distinguish exosomes from heterogenous EVs.^89^ Similar to exosomes, ectosomes also contain a variety of cargos including proteins (e.g., receptor tyrosine kinases, cytosolic signaling proteins) and RNAs such as miRNAs.^87^ Besides, EVs contain various lipids, including sphingomyelin, phosphatidylserine (PS), cholesterol, and ceramide. These lipids are mainly located on the surface of EVs to control their biogenesis.^90^, ^91^

Notably, there is no single optimal EV separation method, and absolute purification of EVs from culture medium or biological fluids has remained an unrealistic goal until now.^88^, ^92^, ^93^ Various EV separation/enrichment protocols have been developed, including ultracentrifugation, density gradient centrifugation, filtration, size‐exclusion chromatography, modified polymer co‐precipitation, and commercially available isolation kits. Each technique has disadvantages that include low purity, high cost, insufficient homogeneity, and high labor intensity.^93^, ^94^, ^95^ For example, methods including precipitation kits/polymer (polyethylene glycol or others), low molecular weight cutoff centrifugal filters with no further separation step, and ultracentrifugation without lower speed steps are inappropriate for EV isolation due to low specificity, according to MISEV 2018 guidelines.^88^ Hence, one or more additional steps following the primary one, such as washing in EV‐free buffer, ultrafiltration, density gradients, or chromatography, are highly recommended to achieve better specificity of EV isolation, even though they may lead to low recovery.^88^

### 3.3 Uptake of EVs

After being released into the extracellular space, EVs can be internalized by various types of brain cells that include NSCs, neurons, astrocytes, and microglia (Figure 2).^23^, ^24^, ^96^ There are three ways for EVs to reach recipient cells, including autocrine, paracrine, and telecrine deliveries. Upon reaching recipient cells, EVs are then internalized by target cells via endocytosis,^97^ phagocytosis,^98^ micropinocytosis,^99^ and lipid‐raft‐mediated endocytosis.^100^ Important factors that participate in the interaction between EVs and the target cells include integrins, lectins, lipids, tetraspanins, heparan sulfate proteoglycans, and extracellular matrix. Although some data are available to describe their interactions, the biological mechanisms by which EVs specifically target recipient cells remain unclear. A study on the interaction of exosomes and dendritic cells (DCs) revealed that milk fat globule‐E8/lactadherin, CD11a, CD54, and PS mediate exosome targeting to DCs.^101^ In addition, heparan sulfate on the surface of target cells can be used as a receptor for exosome uptake, which binds to fibronectin on the surface of exosomes to mediate exosome–cell interaction; removal of heparan sulfate from cell surface leads to fibronectin release and significantly inhibits exosome–target cell interaction.^102^ A study of exosome proteomics also demonstrated that different integrin expression patterns were associated with exosome uptake and knocking down integrins α6β4 and αvβ5 decreased exosome uptake.^103^ Moreover, tetraspanin plays an important role in the selection of exosome target cells. Exosomes that express the Tspan9–CD49d complex preferentially bind to endothelial cells and initiate angiogenesis.^104^ Integrins are important not only for the uptake of exosomes, but also for that of ectosomes. In a study that used MSC‐derived ectosomes to treat acute kidney injury, researchers reported that ectosomes were incorporated into renal tubular cells via CD44 and β1 integrins.^105^ It is worth‐noting that the uptake of EVs is not necessary for exosomes to achieve their biological functions. EVs can modulate intracellular signaling by directly binding to the surface receptors on the recipient cells.^106^ For example, DC‐derived EVs may enhance immune responses of DCs by binding to Toll‐like receptor ligands on bacterial surface,^107^ and activate T lymphocytes through CD40–CD40L interaction,^108^ although whether these EVs are incorporated into target cells after ligand–receptor interaction remains unknown.

## 4 Functions of NSC‐EVs

**FIGURE 4:**
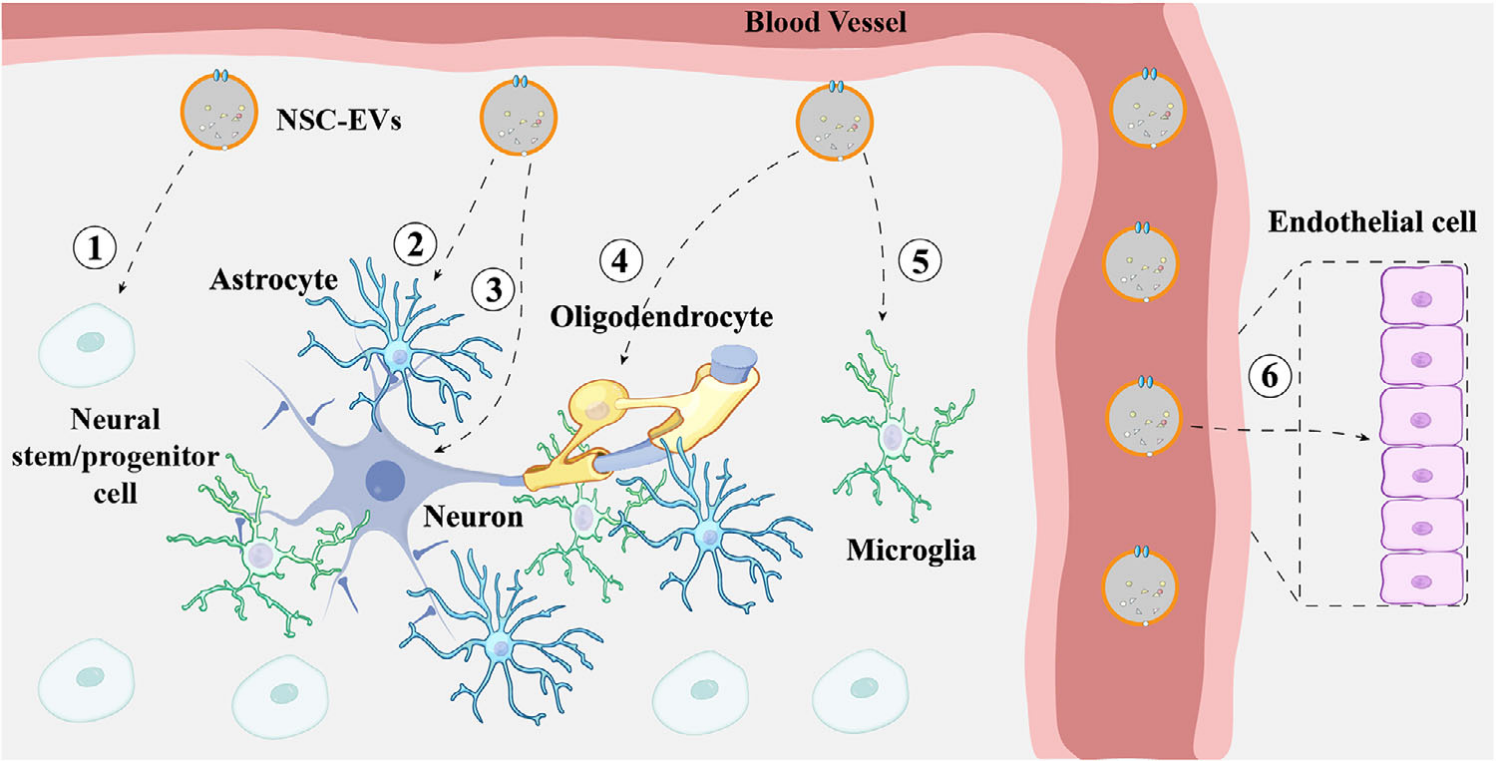
The functions of neural stem cell‐derived extracellular vesicles (NSC‐EVs) in the central nervous system microenvironment. After administration, NSC‐EVs can be internalized by various brain cells and vascular endothelial cells to exert their bio‐functions and therapeutic effects. ① NSC‐EVs promote the proliferation, differentiation, migration, and survival of NSCs after being internalized by NSCs. ② NSC‐EVs inhibit excessive cell activation and promote the release of neurotrophins after being internalized by astrocytes. ③ NSC‐EVs rescue neuronal and synaptic damage from oxidative stress, and promote autophagy after being internalized by neurons. ④ NSC‐EVs promote remyelination after being internalized by oligodendrocytes. ⑤ NSC‐EVs inhibit excessive microglial activation, prevent the release of toxic substances, and regulate cell morphology after being internalized by microglia. ⑥ NSC‐EVs stimulate endothelial cell vascular regeneration, increase the expression of cytokines released by endothelial cells, maintain endothelial cell adhesion and stability, and limit endothelial cell permeability changes caused by external influences after being internalized by vascular endothelial cells.

In recent years, growing evidence has shown that NSCs secrete EVs to modulate various physiological and pathological processes, including neuroinflammation, neuroprotection, BBB integrity maintenance, neuroregeneration and neurogenesis, etc. Here, we summarize current findings on multiple functions of NSC‐EVs in pathological progressions (Figure 4).

### 4.1 Microglial function regulation

Microglia are innate immune cells derived from yolk sac macrophages in the CNS.^109^, ^110^ They play an important role in the phagocytosis and clearance of pathogenic molecules and neuroinflammatory responses.^111^, ^112^, ^113^, ^114^ Macrophages invade the mid‐gestation brain and generate the majority of microglia during the first postnatal weeks.^115^, ^116^ A recent study demonstrated that SVZ NSCs produce and release abundant EVs.^110^ These EVs were primarily internalized by microglia, and induced a transition to a CD11b/Iba1 non‐stellate microglial morphology. Subsequently, microglia release cytokines to control NSC proliferation, therefore forming a negative feedback loop. These findings implicate NSC‐EVs as a microglial morphogen, providing insight into normal and pathophysiological brain development.

Microglia‐driven neuroinflammation is a host defensive mechanism through which CNS protects itself from pathogenic and/or infectious insults. Mild inflammation is a process of systematic response to changed environments in mammalian physiology that protects the body. However, excessive activation of the pro‐inflammatory pathways is pathogenic, leading to multiple pathological changes such as impaired microvascular integrity, excessive pro‐inflammatory cytokine secretion, and massive immune cell recruitment.^117^, ^118^ Consequently, immune cells and immune cell‐released neurotoxic molecules attack neurons and other brain cells, eventually resulting in neuronal damage and death.^119^, ^120^ NSC‐EVs have been reported to effectively inhibit the aggregation of inflammation factors and ameliorate local inflammation in various neurodegenerative diseases.^121^, ^122^ Besides, NSC‐EVs effectively inhibit the activation of CD68^+^ microglia and the morphological changes in microglia, decrease the secretion of inflammation factors, and block the recruitment and aggregation of peripheral immune cells such as monocytes and macrophages in acute neurological diseases such as stroke and lipopolysaccharide‐stimulated neuroinflammation.^23^, ^123^, ^124^ These studies suggest that NSC‐EVs play an important role in the modulation of inflammatory responses in the CNS.

### 4.2 Neuroprotection

Given the dominantly important role of CNS in mammalian body functions, maintaining the homeostasis of CNS microenvironment and protecting the CNS from infection or invasion is crucial to avoid the onset of neurological diseases. Emerging evidence has proven that NSC‐EVs play an important role in neuroprotection. For example, recent studies reported that NSC‐EVs significantly reduced the expression of pro‐apoptotic proteins and increased the expression of anti‐apoptotic proteins to rescue cell death in vitro.^27^, ^125^, ^126^, ^127^, ^128^ Accumulating evidence also reveals that NSC‐EVs protected synaptic functions and maintained impulse conduction.^26^, ^129^, ^130^, ^131^ Moreover, NSC‐EV administration improved the vitality of neurons in animal models of different neurological diseases, which indicates that NSC‐EVs effectively protect neurons in diverse pathological conditions.^28^, ^126^, ^130^ One possible mechanism of NSC‐EV‐mediated neuroprotection is through restoring the balance of oxidation and antioxidant system to alleviate oxidative stress. Multiple studies have reported that NSC‐EVs enhanced the expression of antioxidant enzymes and reduced the production of reactive oxygen species (ROS), which rescued mitochondrial dysfunction and neuronal loss in neurodegenerative diseases.^121^, ^122^, ^130^ NSC‐EVs also activated extracellular signal‐regulated kinase (ERK)‐related signaling pathways, therefore protecting brain cells from oxidative stress‐induced apoptosis.^128^ Besides, NSC‐EVs promoted cell survival by enhancing autophagy, which clears excessive toxic substances in pathological conditions.^123^, ^132^ Taken together, these studies suggest that NSC‐EVs effectively protect CNS microenvironment homeostasis and prevent neuronal dysfunction.

### 4.3 BBB integrity maintenance

The BBB constituted by brain microvascular endothelial cells is a dynamic interface that controls and regulates the transport of diverse substances, including peptides, proteins, ions, vitamins, hormones, and immune cells, between the circulation and the brain parenchyma to maintain CNS homeostasis. Defects in BBB increase the permeability of vascular endothelial cells, which have a great impact on CNS microenvironment.^133^ NSC‐EVs can be taken up in brain endothelial cells via heparan sulfate proteoglycan‐dependent endocytosis.^20^ More importantly, multiple studies have reported that NSC‐EVs effectively maintain BBB integrity in different disease models. For example, NSC‐EVs have been reported to protect spinal cord microvascular endothelial cells (SCMECs) and increase the expression of adhesion proteins in hypoxic environments.^27^, ^134^ Similarly, Zhang et al.^124^ found that NSC‐EVs enhanced post‐stroke BBB integrity via mitigating upregulation of ABCB1 and matrix metalloproteinase 9 and activation of the nuclear factor‐kappa B (NF‐κB) pathway using an in vitro BBB co‐culture model. NSC‐EV treatment also increased the node number and total branch length of human umbilical vein endothelial cells (HUVECs) and enhanced the migration capacity of HUVECs.^130^ Moreover, NSC‐EVs protect endothelial cell integrity, stimulate endothelial cell vascular regeneration, and increase the production and release of cytokines by endothelial cells.^29^, ^135^ Taken together, NSC‐EVs serve as a dominant modulator for the permeability of the BBB in physiological and pathological conditions, and the administration of NSC‐EVs is a promising therapeutic strategy for BBB injury and dysfunction.

### 4.4 Neuroregeneration and neurogenesis

Neuroregeneration is a complicated process that involves the expansion of adult NSC pool, neurogenesis for generation of desired types of neurons, regrowth of damaged axons, and re‐establishment of interrupted neural pathways/circuits under pathological conditions. Although neurogenesis in adulthood is significantly reduced compared with that in the fetus stage, there are still some NSCs residing in the SGZ and SVZ. These NSCs differentiate into mature neurons and migrate to functional areas where they eventually integrate into existing functional neural networks. Growing evidence has shown that NSC‐EVs play an important role in regulating neuroregeneration and neurogenesis. We and other independent groups demonstrated that NSC‐EVs promote the differentiation of NSCs into both neuronal and glial lineages in vitro presumably by transferring miR‐9 and miR‐21 to inhibit Notch signaling pathway in target cells.^136^, ^137^, ^138^, ^139^ NSC‐EVs could also effectively increase adult neurogenesis in the dentate gyrus (DG) area of hippocampus of insulin‐resistant mice.^140^ Similar results have been found in multiple sclerosis (MS) and ischemic stroke models in which NSC‐EVs effectively expand the NSC pool and promote neurogenesis to replace injured neurons.^24^, ^129^ Surprisingly, NSC‐EVs promote MSC differentiation into functional neurons.^141^ Derkus et al.^141^ cocultured rat SVZ NSCs with human MSCs and found that NSC‐EVs switched hMSCs into neuroglial cells or NSC‐like cells. In line with this finding, Ma et al.^142^ reported that NSC‐EVs promoted neuronal differentiation of MSCs probably by regulating Netrin1. Together, these studies provide important insights into the positive effects of NSC‐EVs on neuroregeneration and neurogenesis.

### 4.5 Others

Except for the aforementioned positive roles, NSC‐EVs display other functions such as regulating the entry of viruses, transporting functional mitochondria, and changing the morphology of microglia. In 2014, Sims et al.^143^ reported that NSC‐EVs significantly facilitated type 5 adenovirus (Ad5) entry into Coxsackie virus and adenovirus receptor (CAR)‐deficient cells, which could be blocked by treatment with anti‐T‐cell immunoglobulin mucin protein 4 (TIM‐4). This finding indicates that NSC‐EVs play an important role in mediating the entry of the virus into cells. Moreover, Peruzzotti‐Jametti et al.^144^ reported that NSC‐EVs traffic functional mitochondria to rescue mitochondrial functions in recipient cells. NSC‐EVs exhibited therapeutic effects, including improving the survival rate of Rho° cells, restoring the dynamics of mitochondria and cell metabolism, and ameliorating the excessive generation of pro‐inflammatory molecules in both mtDNA‐deficient L929 Rho° cells and MS animals.^144^

Overall, current evidences support that NSC‐EVs exert comprehensive functions in neuroprotection, neuroinflammation, BBB integrity maintenance, and neuroregeneration and neurogenesis, suggesting that NSC‐EVs may serve as novel therapeutics of neurological diseases.

## 5 Application of NSC‐EVs IN NEUROLOGICAL DISEASES AND OTHER DISEASES

As discussed above, NSC‐EVs effectively pass through the BBB and escape from the phagocytosis of mononuclear phagocytes and degradation due to their small size and low immunogenicity.^145^, ^146^, ^147^ NSC‐EVs are also much less neurotoxic than nano‐drugs, avoiding the risk of NSC transplantation‐induced tumor formation.^148^, ^149^ More importantly, NSC‐EVs displayed promising neuroprotective, anti‐inflammatory, and neuroregenerative effects, making them potential therapeutics of neurological diseases (Table 2).^150^

**TABLE 2:** Summary of the application and function of neural stem cell‐derived extracellular vesicle (NSC‐EV) in neurological diseases and other diseases

Disease	Animal/cell model	NSC‐EV type	EV isolation^a^	Cargos	Targets	Function	Ref.
AD	5×FAD mice model	hNSC‐EV	Filtration (0.22 μm), ultracentrifugation	miR‐125‐5p, miR‐124, 3p, miR‐125a‐5p	–	Improve cognitive function, reduce Aβ plaque, inhibit neural and peripheral inflammation, restore synaptophysin expression	^26^
	APP/PS1 mice model	mNSC‐EV	Filtration (0.22 μm), ultracentrifugation	–	SIRT1	Improve cognitive and synaptic function, inhibit inflammation, and microglial activation	^152^
PD	SH‐SY5Y cells, 6‐OHDA‐induced PD mice model	F3‐NSC‐EV	Centrifugation, filtration (0.22 μm), commercial EV isolation kit	hsa‐mir‐17, hsa‐mir‐20a, hsa‐mir‐183‐5p	–	Reduce neurotoxicity and oxidative stress, repress pro‐inflammatory cytokine production and neuroinflammation, protect dopaminergic neurons	^122^
RD	RCS mice model	hNSC‐EV	Density gradient, ultracentrifugation	Let‐7a‐5p, miR‐26a‐5p, miR‐21‐5p	TNF‐α, IL‐1β, COX‐2	Inhibit microglial activation, delay photoreceptor degeneration, preserve visual function, prevent thinning of the outer nuclear layer, repress apoptosis of photoreceptors	^25^
IS	Endothelial cell, OGD cell model, MCAO mice model	mNSC‐EV	PEG precipitation, ultracentrifugation	–	–	Inhibit NF‐κB pathway and inflammatory cell recruitment, protect BBB integrity, reduce BBB leakage	^124^
	OGD cell model, MCAO mice model	SVZ‐NSC‐EV	Ultracentrifugation	miR‐20a, miR‐26b, miR‐124	–	Protect brain cell death, reduce post‐ischemic motor dysfunction, stimulate nerve regeneration and synaptic plasticity, reversing immunosuppression	^159^
	MCAO rat model	rNSC‐EV	Ultracentrifugation	–	–	Reduce ischemic lesion area, improve motor activity, restore exploratory behavior, inhibit microglial activation	^28^
	H9 cells, MCAO mice model	H9‐NSC‐EV	Filtration (0.22 μm), ultrafiltration	–	–	Improve neurological function, promote exercise capacity, inhibit systemic inflammation, enhance peripheral immune response, reduce ischemic obstruction area	^158^
	SH‐SY5Y cells, MCAO rat model	rNSC‐EV	Filtration (0.22 μm), ultracentrifugation	miR‐150‐3p	Caspase2	Reduce infarct size, inhibit neuronal apoptosis	^125^
	MCAO pig model	mNSC‐EV	Filtration (0.22 μm), ultrafiltration	–	–	Decrease lesion volume, mitigate cerebral swelling, promote blood diffusive, sustain integrity of white matter, increase motor activity, restore locomotive	^29^
SCI	SCI rat model, SCMECs, cell hypoxia model	FTY720‐NSC‐EV	Filtration (0.22 μm), ultrafiltration	–	–	Improve motor function, prevent spinal cord edema formation, reduce SCI lesion area, inhibit edema‐ and apoptosis‐related protein expression, protect SCMECs	^27^
	SCMEC cells,	mNSC‐EV	Filtration (0.22 μm), ultrafiltration	–	VEGF‐A	Promote angiogenesis, promote SCMECs proliferation and migration, accelerate microvascular and spinal cord regeneration, reduce lesion area	^134^
	SCI rat model, PC 12 cells, primary neuron	mNSC‐EV, IGF‐NSC‐EV	Filtration (0.22 μm), ultrafiltration	miR219a‐2‐3p	YY1	Promote regeneration, reduce lesion damage area, promote functional recovery, inhibit neuronal apoptosis	^126^
	SCI rat model	SVZ‐NSC‐EV	Ultracentrifugation	–	–	Inhibit inflammation gene expression, improve locomotive recovery, protect neuron morphology	^135^
TBI	mTBI rat model	hNSC‐EV	Commercial EV isolation kit	–	–	Modify neurological severity score, inhibit reactive astrocytes, promote neurogenesis	^165^
	CCI‐induced TBI rat model	H9‐NSC‐EV	Filtration (0.22 μm), ultrafiltration	–	–	Reduce lesion sizes, enhance presence of endogenous NSCs, attenuate motor function.	^166^
HAND	SHSY5Y cells, CCF0STTG1 cells, TIB‐202 cells, CCL‐185 cells	ATCC ACS‐5003‐EV	Ultracentrifugation	AC120498.9	ADIRF‐AS1, AC120498.9	Protect the morphology and function of HIV‐infected neurons, inhibit inflammatory factor expression	^127^
VD	HNCs, VD rat model	hNSC‐EV	Ultracentrifugation	miR‐34b‐5p	CALB1	Block the secretion of oxidative stress and inflammatory factors, reverse the damage of hippocampal neurons, improve learning ability and memory	^121^
RICD	Radiation mice model	hNSC‐EV	Ultracentrifugation	miR‐124‐3p	–	improve exercise and fear behavior, reverse cognitive impairment, reduce microglia overactivation	^183^
	ATN rat model, radiation mice model	hNSC‐EV	Ultracentrifugation	–	–	Maintain irradiated neurons structural plasticity, enhance the expression of neurotrophic factor and synaptic signaling protein, reduce radiation‐induced neuroinflammation	^131^
Insulin resistance	IPA‐induced IR mice model, Sh‐SY5Y cells	mNSC‐EV	Commercial EV isolation kit	–	–	Restore NSC proliferation, delay NSC senescence, activate FoxO transcription, facilitate hippocampal neurogenesis	^140^
Myocardial infarction	Myocardial ischemia mice model	CTX0E03‐hNSC‐EV	Filtration (0.22 μm), size‐exclusion chromatography	–	Gp130/JAK	Reduce infarct size, delay ROS‐mediated mPTP open	^190^
NEC	NEC mice model	AF‐NSC‐EV, E‐NSC‐EV	Ultracentrifugation	–	–	Reduce incidence and severity of NEC, suppress intestinal damage	^188^

Abbreviations: Aβ, β‐amyloid; AD, Alzheimer's disease; AF‐NSC‐EV, amniotic fluid‐derived neural stem cell‐derived extracellular vesicle; ATN, athymic nude; BBB, blood–brain barrier; CCI, controlled cortical impact; E‐NSC‐EV, neonatal enteric neural stem cell‐derived extracellular vesicle; FoxO, Forkhead box O; H9‐NSC‐EV, H9 cell‐induced neural stem cell‐derived extracellular vesicle; HAND, HIV‐associated neurocognitive disorders; HNCs, hippocampal neuronal cells; hNSC‐EV, human neural stem cell‐derived extracellular vesicle; IGF‐NSC‐EV, insulin‐like growth factor‐1‐neural stem cell‐derived extracellular vesicle; IL, interleukin; IPA, insulin and palmitic acid; IS, ischemic stroke; MCAO, middle cerebral artery occlusion; mNSC‐EV, mouse neural stem cell‐derived extracellular vesicle; mPTP, mitochondrial permeability transition pore; NEC, necrotizing enterocolitis; NF‐κB, nuclear factor‐kappa B; NSC, neural stem cell; NSC‐EV, neural stem cell‐derived extracellular vesicle; OGD, oxygen glucose deprivation; PD, Parkinson's disease; PEG, polyethylene glycol; RCS, Royal College of Surgeons; RD, retinal degeneration; RICD, radiation‐induced cognitive dysfunction; rNSC‐EV, rat neural stem cell‐derived extracellular vesicle; ROS, reactive oxygen species; SCI, spinal cord injury; SCMECs, spinal cord microvascular endothelial cells; SVZ‐NSC‐EV, subventricular zone‐neural stem cell‐derived extracellular vesicle; TBI, traumatic brain injury; TNF, tumor necrosis factor; VD, vascular dementia; VEGF‐A, vascular endothelial growth factor‐A.

^a^ All methods of EV isolation are with low‐speed centrifugation unless otherwise noted.

### 5.1 NSC‐EVs and neurodegenerative diseases

Neurodegenerative diseases are a class of refractory diseases caused by multifactor‐induced neurodegeneration. With the extension of human lifespan, the incidence of neurodegenerative diseases has increased year by year, creating great medical, social, and economic burdens worldwide. In recent years, some studies have shown that EVs bring promising therapeutic effects on cellular and animal models of neurodegenerative diseases, such as AD, PD, MS, and retinal degeneration (RD).

AD is the most common neurodegenerative disease and number one cause of dementia in the elderly worldwide.^151^ Extracellular aggregation of β‐amyloid (Aβ) is the pathological hallmark and considered to be the direct cause of AD. Apodaca et al.^26^ isolated EVs from the conditioned medium of human embryonic stem cell‐derived NSCs through filtration (0.22 μm) and ultracentrifugation. Retro‐orbital vein injection of NSC‐EVs restored fear extinction memory consolidation and reduced anxiety‐related behaviors of 5×FAD mice, and effectively reduced Aβ aggregation and microglia‐driven neuroinflammation.^26^ The therapeutic effects of NSC‐EVs on AD were further confirmed by Li et al.^152^ NSC‐EVs significantly upregulated the expression levels of growth‐associated protein 43, synaptophysin, postsynaptic density 95 (PSD95), and microtubule‐associated protein 2 to prevent synaptic loss, and dramatically reduced those of oxidative damage markers such as anti‐4‐hydroxynonenal and anti‐3‐nitrotyrosine, inflammatory cytokines, and microglial markers to repress mitochondrial dysfunction and neuroinflammation in the brains of 5×FAD mice, suggesting that NSC‐EVs may serve as a potential therapeutics for AD.^26^

PD is one of the most disabling CNS disorders and the second most common progressive neurodegenerative disease in the elderly.^153^ Excessive ROS‐induced mitochondrial dysfunction and microglial activation have emerged as essential contributors to the degeneration of dopaminergic neurons in PD pathogenesis.^154^ Recent studies collected EVs from conditioned medium of a human fetal telencephalon‐derived immortal NSC line, F3 cells, by the combination of centrifugation, filtration, and EV isolation kit.^122^ NSC‐EV treatment attenuated oxidative stress‐stimulated inflammatory responses, therefore protecting neuronal cells from degeneration induced by neurotoxin 6‐hydroxydopamine (6‐OHDA) in vitro and in vivo.^122^ Moreover, NSC‐EVs are highly enriched with miR‐9, let‐7, miR‐17, miR‐183‐5p, and miR‐20a that are involved in the regulation of neuroregeneration and neuroinflammation. These miRNAs target Forkhead box O (FoxO)1 and FoxO3 to facilitate neurite regeneration and prevent oxidative stress‐induced apoptosis, leading to the alleviation of PD phenotypes.^122^

RD is a common neurodegenerative disease characterized by progressive degeneration of photoreceptors. In recent years, NSC transplantation has become a promising therapy for RD, but the underlying mechanisms remain unclear. Bian et al.^25^ collected NSC‐EVs via sucrose density gradient separation and ultracentrifugation, and injected them into the subretinal space of the Royal College of Surgeons rats, an RD model. Results showed that NSC‐EVs significantly inhibited microglial activation and protected photoreceptors from apoptosis, suggesting that NSC‐EVs are a key factor that mediates the therapeutic effects of transplanted NSCs on RD.

Taken together, previous studies have demonstrated that NSC‐EVs are promising therapeutics for neurodegenerative diseases. However, we are still far away from fully understanding the underlying mechanisms of NSC‐EV‐based therapy. The horizontal delivery of RNAs, particularly non‐coding RNAs such as miRNAs, within NSC‐EVs to directly inhibit the expression of disease‐related genes may be one mechanistic pathway, which has been reported by multiple studies.^25^, ^122^ However, since most current neurodegenerative disease models only present certain pathological features but are unable to mimic disease pathogenesis, the results generated using these models may fail to reflect the therapeutic effects of NSC‐EVs on patients. In summary, more efforts are urgently needed to overcome these concerns to push NSC‐EVs to clinical practice in the future.

### 5.2 NSC‐EVs and acute neurological diseases

Compared with therapeutics for neurodegenerative diseases, acute neurological disease treatment mainly focuses on reducing neuroinflammation and enhancing neuroprotection and neuroregeneration. Given that NSCs play an important role in neural tissue repair and regeneration, NSC‐EVs are regarded as a promising natural resource for the treatment of acute neurological diseases.^155^ Recent studies have revealed that NSCs may exert a therapeutic role by secreting EVs that carry neurotrophic factors.^156^

IS is the second leading cause of death in the world and NSC‐EV treatment strategies give a promising insight for preventing neural injury during reperfusion. It has been shown that NSC‐EVs effectively restore motor activity and inhibit apoptosis of neurons in vivo and in vitro.^125^ Similar results have been obtained in multiple rodent models that mimic ischemic conditions.^28^, ^157^ The therapeutic effects of NSC‐EVs are mainly on the modulation of immune responses. Webb et al.^158^ showed that NSC‐EVs effectively reduced secondary damage caused by cascade reactions of IS, including the promotion of M2 macrophage phenotype transition and the reduction of Treg and Th17 populations in a middle cerebral artery occlusion (MCAO)‐induced rodent IS model. Similarly, NSC‐EVs reversed oxygen glucose deprivation‐induced inflammatory activity of neurons presumably by activating mTOR signaling.^157^ However, the immunomodulatory effects of NSC‐EVs are highly dynamic with finite duration since conflicting results were reported by Zheng et al.^159^ that NSC‐EVs did not affect leukocytes (CD45^high^), monocytes (CD45^high^CD3^−^CD11b^+^), B cells (CD45^high^CD3^−^CD19^+^), or T cells (CD45^high^CD3^+^) in a mouse stroke model. Instead, NSC‐EVs reversed post‐ischemic peripheral immunosuppression as they elevated the expression levels of B and T lymphocytes in the blood.^159^ Under hypoxic conditions, NSCs also release miR‐120‐enriched EVs to enhance cell viability.^160^ Bioinformatic analyses further revealed that miR‐98‐3p was a key component of hypoxic preconditioning, through which NSC‐EVs upregulated the activities of phosphoinositide‐3‐kinase (PI3K)/protein kinase B (AKT), mitogen‐activated protein kinase (MAPK), mammalian target of rapamycin (mTOR), and endocytosis signaling pathways to facilitate cell survival in a stroke therapy.^161^ In addition, NSC‐EVs significantly reduced the infarction area and suppressed neuronal death via horizontal delivery of miR‐150‐3p to neurons to target CASP2, confirming the anti‐apoptotic effects of NSC‐EVs in a MCAO‐induced rodent IS model.^125^ The therapeutic potential of NSC‐EVs was further evaluated using a porcine model of ischemic stroke.^29^ Webb et al. enriched NSC‐EVs by filtration (0.22 μm) and ultrafiltration, and EVs were administered via peripheral ear vein. NSC‐EV treatment led to increased motor activity and exploratory behavior, together with faster and improved recovery of spatiotemporal gait parameters. Importantly, Webb et al.^158^ also compared the therapeutic effects of NSC‐EVs and MSC‐EVs and found that NSC‐EVs exerted better outcomes on reducing neural damage and augmenting the systemic immune response, compared with MSC‐EVs in a murine thromboembolic stroke model. This finding is corroborated by other studies that demonstrate the inhibitory roles of NSC‐EVs, but not MSC‐EVs, in neuroinflammation and systemic inflammation, suggesting outstanding therapeutic potential of NSC‐EVs for stroke.^24^, ^30^

Acute traumatic SCI is a disabling event without effective treatment until now. It is characterized by irreversible motor dysfunction and even death. Due to the fading plasticity of SCMECs, patients gradually lose self‐care ability after injury. Disability places heavy burdens on patients, their families, and the society.^162^ Over the past decades, several studies reported that NSC‐EVs provided a promising insight for treating SCI. Zhong et al. reported that NSC‐EVs contained high levels of vascular endothelial growth factor‐A to increase SCMECs’ angiogenesis, promote blood vessel repair, and reduce necrotic bone marrow cavity after spinal cord injury in vivo.^134^ Interestingly, another SCI study indicated that NSC‐EVs loaded with FTY720, a functional antagonist of sphingosine 1‐phosphate receptor‐1, drove microvascular remodeling and regenerated bone tissue by activating phophatase and tensin homolog (PTEN)/AKT signaling pathway.^27^ Except ameliorating injury by promoting angiogenesis, growing evidence has shown that NSC‐EVs attenuate SCI‐induced apoptosis and neuroinflammation.^126^ Ma et al.^126^ have demonstrated that NSC‐EVs upregulated miR‐19a‐2‐3p to inhibit YY1 expression and NF‐κB pathway, thus inhibiting neuroinflammation and promoting neuroprotection. Similar results were obtained by Rong et al.^132^ who found that NSC‐EVs inhibited neuroinflammation, reduced neuronal apoptosis, and promoted functional recovery, likely by activating autophagy in an SCI rat model. Another study from this team further confirmed that NSC‐EVs delivered 14‐3‐3t to enhance autophagy by targeting Beclin‐1, therefore reducing SCI‐induced cell death and neuroinflammation. These findings provide new insights and theoretical basis for treating SCI with NSC‐EVs.^123^

Traumatic brain injury (TBI) is the third leading cause of death all over the world.^163^ It is caused by one or more attacks with severe mechanical forces on the brain and leads to a series of neurological function changes.^164^ In recent years, EV‐based stem cell‐free therapies have been investigated in the course of TBI treatment. Abedi et al.^165^ reported that administration of NSC‐EVs improved recent‐period neurological behaviors and promoted neurogenesis after TBI. Similarly, another study showed that NSC‐EVs significantly reduced lesion sizes, enhanced presence of endogenous NSCs, and attenuated motor function impairments in a TBI rodent model.^166^ Thus, both studies revealed promising neuroprotective and functional benefits of NSC‐EVs for treating TBI.

Emerging evidence has implied the potential promise of NSC‐EVs as a therapeutic strategy for acute neurological diseases. However, it is noteworthy that there is still a large gap between current knowledge and the complexity of disease pathogenesis. If we would like to translate NSC‐EV‐based therapy from bench to bed, we urgently need more detailed work to investigate factors such as safety, time window, and dose–response.

### 5.3 NSC‐EVs and dementia/cognitive dysfunction

Dementia is a syndrome characterized by cognitive decline that is significant enough to interfere with patients’ independent, daily functioning.^167^ With the exacerbation of population aging, dementia/cognitive impairment has emerged as a nonneglected problem worldwide. In some Western countries with a heavy load of aged population, cognitive dysfunction is listed as the major health challenge. Patients were affected from many aspects by cognitive impairment, including attention,^168^ long‐term memory,^169^ processing speed,^170^ working memory,^171^ and executive function.^172^ Beside classic neurodegenerative diseases such as AD, dementia/cognitive dysfunction can be caused by many other pathogenies, including nutrient excess, vascular disorder, human immunodeficiency virus (HIV) infection, and radiation exposure. Due to the complexity of their pathogenesis, it is hard to find an ideal treatment option for dementia/cognitive dysfunction.

Over the past decades, with the development of EVs, emerging evidence suggests that NSC‐EVs exhibit promising therapeutic effects on dementia/cognitive dysfunction. In 2020, Spinelli et al.^173^ found that after intranasal administration of NSC‐EVs, the transcription of BDNF was epigenetically restored in high‐fat diet (HFD)‐fed mice. BDNF regulation of synapses has emerged as one of the most important mechanisms in the pathogenesis of cognitive impairment‐related diseases.^174^ Long‐term potential (LTP) that is essential for memory formation, storage, and forgetting is regulated by the expansion and remodeling of the PSD and extension of pre‐existing dendritic spines that require synthesis of synaptic proteins.^175^, ^176^ BDNF is stored at glutamatergic synapses and acts as the trigger for protein synthesis‐dependent late LTP.^177^, ^178^, ^179^ Hence, given the pivotal role of BDNF in synaptic plasticity and neural circuit regulation, elevated BDNF expression abrogated HFD‐induced memory impairment.^173^, ^174^, ^180^, ^181^ Furthermore, the intranasal administration of NSC‐EVs counteracted HFD‐dependent impairment of neurogenesis in the hippocampus of adult mice by activating insulin receptor substrate‐1/FoxO signaling cascade and inhibiting the recruitment of FoxO1 and FoxO3a on the promoters of genes that regulate proliferation and self‐renewal of NSCs.^140^

Vascular dementia (VD) is the number two cause of dementia after AD, causing around 15% of cases.^182^ To date, no treatment has been licensed for VD, indicating the importance and urgency to develop novel effective therapeutic strategy for treating VD. Recently, Qi et al.^121^ applied NSC‐EVs on a VD rat model, and their results showed that NSC‐EVs reduced the pyramidal cell layer necrosis, mitigated oxidative stress, and repressed inflammatory factor secretions. Moreover, the therapeutic effects of NSC‐EVs on VD are possibly mediated by the delivery of lncRNA myocardial infarction association transcript that regulates miR‐34b‐5p/CALB1 axis.^121^ Their study provided evidence to support the feasibility of NSC‐EV‐based therapy.

Radiation‐induced cognitive dysfunction is increasingly recognized as an important risk for cranial radiation therapy. NSC‐EVs, collected by ultracentrifugation, have displayed promising therapeutic effects on radiation‐induced cognitive dysfunction (RICD).^131^, ^183^ One key pathological feature of RICD is the abnormal expression of PSD95, an important postsynaptic scaffold protein and downstream molecule of BDNF, which causes alterations in synaptic integrity and impairment to synaptic function.^184^ NSC‐EV treatment reduced the expression of PSD95, rescued dendritic spine density, and ameliorated abnormal microglial activation in the DG of the hippocampus in RICD mice.^131^ Moreover, Leavitt et al.^183^ found that NSC‐EVs inhibited microglial activation and attenuated RICD probably through the delivery of neuroprotective miRNA, miR‐124, confirming the therapeutic potential of NSC‐EVs in RICD.

HIV‐associated neurocognitive disorders (HAND) affect at least 50% of HIV‐1‐infected individuals.^185^ Although the exact pathogenesis of HAND remains poorly understood, the excessive activation of inflammatory pathways has emerged as a primary response that contributes to the neurological damage in HAND.^186^ Utilizing human‐induced pluripotent stem cell (iPSC)‐derived NSCs, Branscome et al.^127^ generated an in vitro HIV‐infection model. They reported that iPSC‐EVs displayed the neuroprotective and anti‐inflammatory effects on HIV‐1‐damaged NSC‐derived neurosphere cells and SHSY5Y neuronal cells.^127^ Given that iPSC‐derived NSCs exhibited much greater neurogenic potential than iPSCs, this study highlighted the therapeutic potential of NSC‐EVs for rescuing neuronal damage induced by HIV‐1 infection, although rigorous experimental validation and confirmation are required.

In summary, recent studies have revealed encouraging findings in using NSC‐EVs for treating dementia/cognitive dysfunction. However, more comprehensive investigations are urgently required to validate the safety and efficacy of NSC‐EV‐based therapies in other types of dementia in animal models and to promote the translation of basic research results to clinical practice.

### 5.4 NSC‐EVs and peripheral diseases

Interestingly, emerging evidence has demonstrated the involvement of NSC‐EVs in the treatment of various diseases in the peripheral system, such as necrotizing enterocolitis (NEC) and myocardial infarction.

As a complication of premature delivery, NEC has an etiology that is not fully understood by clinicians.^187^ Inspiringly, NSC‐EVs have exhibited convincing therapeutic effects on NEC. McCulloh et al.^188^ isolated EVs from amniotic fluid and neonatal enteric NSCs via five rounds of (ultra)centrifugations and treated NEC animals with NSC‐EVs by a single intraperitoneal injection. The incidence of NEC decreased from 60.7% to 11.1% in NSC‐EV‐treated animals compared with control subjects, which indicates that NSC‐EVs are an effective therapy for NEC. More importantly, the authors also compared the therapeutic potential of NSC‐EVs and MSC‐EVs, and the results indicated comparable reductions in the incidence of experimental NEC after the injection of NSC‐EVs and MSC‐EVs.^188^

In acute myocardial infarction and reperfusion, oxidative stress substantially contributes to cardiac damage.^189^ Excess ROS in mitochondria results in the opening of mitochondrial permeability transition pore (mPTP), causing cardiomyocyte death. Therefore, preventing excess mPTP opening is one important aspect of therapies for reducing infarct size. Recently, Katsur et al.^190^ found that NSC‐EVs played an important role in protecting myocardium. NSC‐EVs acted on mPTP and activated gp130/Janus kinase (JAK)/signal transducers and activators of transcription (STAT) pathway to delay mPTP opening, therefore effectively avoiding the damage to mPTP by ROS, protecting the mitochondrial function of myocardial cells, and reducing the area of myocardial infarction.^190^

It is noteworthy that, in these studies, NSCs were harvested/generated from different sources, including amniotic fluid,^187^ neonatal enteric NSCs,^187^ and CTX0E03 neuronal stem cells.^190^ The different cell origins of NSCs may release EVs with distinct functions, making the standardization of NSC‐EV generation, characterization, storage, and administration a big concern for clinical applications. In addition, therapeutic effects of NSC‐EVs should also be comprehensively compared with those of MSC‐EVs and EVs derived from other types of stem cells in diverse disease models to further evaluate their therapeutic potential in neurological and peripheral diseases.

## 6 NSC‐EVs AS A DRUG DELIVERY PLATFORM FOR TREATING NEUROLOGICAL DISEASES

Apart from exerting therapeutic effects themselves, NSC‐EVs have been investigated as a potential drug delivery platform in the treatment of neurological diseases. In this section, we summarize studies that used NSC‐EVs as drug delivery vehicles for the treatment of MS, ischemia/hypoxia post‐stroke, and RICD (Table 3).

**TABLE 3:** Summary of the application of neural stem cell‐derived extracellular vesicles (NSC‐EVs) as drug delivery platforms for treating neurological diseases

Disease	Animal/cell model	NSC‐EV type	EV isolation	Cargos	Targets	Function	Ref.
MS	Cuprizone‐induced demeyelination mice model	PS‐Mon‐EV	Ultracentrifugation without low‐speed steps	Monterlukast	GPR17	Suppress demyelination, promote OPCs differentiation, protect axon function, reduce inflammatory factor production, increase neurotrophic factor expression	^193^
	Cuprizone‐ induced demeyelination mice model	PS‐Bryo‐EV	Filtration (0.22 μm), ultracentrifugation	Bryostatin‐1	PKC	Facilitate remyelination, block astrocyte overproliferation and axon injury, inhibit microglial activation	^129^
IS	MCAO mice model	hNSC‐EV	Filtration (0.22 μm), ultracentrifugation	BDNF	–	Improve neurological function, inhibit neuroinflammation, promote neurogenesis	^194^
	BV2 cells, HEK293T cells, MCAO mice model	RGD‐ReN‐EV, ReN‐EV	Filtration (0.22 μm), ultracentrifugation	let‐7g‐5p, miR‐99a‐5p, let‐7i‐5p, miR‐139‐5p, miR‐98‐5p, miR‐21‐5p, let‐7b‐5p	MAPK signaling pathway	Inhibit excessive production of inflammatory factors, reduce infarct size	^23^
Glioma	Glioma cells, glioma‐implant mice	hNSC‐EV	Filtration (0.22 μm), commercial EV isolation kit	miR‐124‐3p	FLOT2	Inhibit glioma growth	^195^
	Glioma cells, glioma‐implant mice	hNSC‐EV	Filtration (0.22 μm), ultracentrifugation	STAT3 antisense oligonucleotides	STAT3	Inhibit glioma growth	^196^

Abbreviations: BDNF; brain‐derived neurotrophic factor; hNSC‐EV, human neural stem cell‐derived extracellular vesicle; IS, ischemic stroke; MCAO middle cerebral artery occlusion; MS, multiple sclerosis; OPCs, oligodendrocyte progenitor cells; PDGFα, platelet‐derived growth factor receptor α; PS‐Bryo‐EV, PDGFα‐decorated bryostatin‐1‐loaded extracellular vesicles; PS‐Mon‐EV, PDGFα‐decorated montelukast‐loaded extracellular vesicles; ReN‐EV, ReNcell ventral mesencephalon‐derived (VM) cell‐derived extracellular vesicle; RGD‐ReN‐EV, arginine‐glycine‐aspartic acid‐ReNcell VM cell‐derived extracellular vesicle.

MS is a chronic autoimmune neurodegenerative disease. The accumulation of a large number of platelet‐derived growth factor receptor α (PDGFRα)‐positive oligodendrocyte progenitor cells (OPCs) in the lesion area of myelin injury is the main pathological feature of MS.^191^ Therefore, promoting the differentiation of OPCs has become a promising therapeutic strategy for remyelination.^192^ Wu et al.^129^ collected EVs from culture medium of bone marrow‐derived NSCs via filtration (0.22 μm) and ultracentrifugation, and utilized NSC‐EVs to deliver anti‐demyelinating drugs montelukast and bryostatin‐1 to OPCs through intravenous injection. These engineered NSC‐EVs improved the protective ability of the myelin sheath, promoted remyelination, blocked astroglial and microglial activation, and mitigated axonal injury.^129^ Xiao et al.^193^ also demonstrated that PDGFA‐ and montelukast‐loaded NSC‐EVs attenuated the process of demyelination and promoted remyelination in vivo, confirming the promising therapeutic effects and drug delivery potential of NSC‐EVs on MS in animal models. However, we should note that NSC‐EVs were harvested only by ultracentrifugation in this study,^193^ which inevitably affects the strength of the conclusion.

Therapeutic strategies for treating ischemia/hypoxia after stroke are extremely limiting. Inspiringly, NSC‐EVs preloaded with potential drugs for ischemia/hypoxia exert promising therapeutic effects on in vitro and in vivo stroke models. For instance, NSC‐EVs loaded with BDNF alleviated neurological deficits and brain damage in MCAO mice post‐stereo‐tactical insertion, accompanied by inhibited microglial activation and promoted neurogenesis.^194^ Besides, surface engineered NSC‐EVs have also been applied to the treatment of stroke. Tian et al.^23^ constructed a recombinant fusion protein that contained arginine‐glycine‐aspartate (RGD)‐4C peptide (ACDCRGDCFC) fused with PS binding domain of lactadherin (C1C2). RGD peptide is able to bind to EV membrane to form RGD‐EVs, which could be used as an outstanding drug delivery platform for stroke therapy by targeting integrin αvβ3.

NSC‐EVs have also been utilized for drug delivery in animals with brain tumors. Qian et al.^195^ loaded miR‐124‐3p into NSC‐EVs via electroporation, and found that engineered EVs inhibited glioma growth in vitro and in vivo by targeting FLOT2. Adamus et al.^196^ also utilized NSC‐EVs to deliver antisense oligonucleotides of STAT3, a promoter of tumorigenesis and resistance to therapies, to microglia to stimulate the immune responses of the latter. Consequently, activated glioma‐associated microglia exhibited enhanced antitumor effects against GL261 glioma in mice.

Thus, numerous studies have demonstrated promising therapeutic outcomes of NSC‐EV‐based drug delivery strategies for MS, IS, and glioma in various cell and animal models, implicating NSC‐EVs as a clinically relevant strategy to improve delivery and safety of therapeutics for neurological diseases.

## 7 CONCLUSIONS AND FUTURE DIRECTIONS

In this review, we summarized the function of NSC‐EVs and their applications in CNS neurological and peripheral diseases. Under physiological conditions, NSC‐EVs play a wide range of regulatory roles in CNS development and function maintenance. In neurological diseases, including neurodegenerative disease, acute neural injury, and dementia‐related disorders, NSC‐EVs and their pivotal cargoes (e.g., BDNF and miRNAs) effectively attenuate oxidative stress, inhibit neuroinflammation, suppress neuronal apoptosis, promote neurogenesis to replace the aging and apoptotic neurons, and restore synaptic structure and functions, leading to a significant delay in disease progression. NSC‐EVs also exhibit therapeutic effects on other system diseases through mechanisms such as effectively protecting the myocardial structure and function, accelerating hair growth, and maintaining the balance in the gastrointestinal microenvironment.

Although NSC‐EVs appear to function like ideal therapeutic drugs or drug delivery platforms for various neurological and peripheral diseases, urgent problems exist in both the basic research and clinical applications of NSC‐EVs. Firstly, it is important to standardize the cell source, generation process, and composition of NSC‐EVs to minimize side effects brought about by the heterogeneity of NSC‐EVs. Secondly, since EVs consist of exosomes, ectosomes, and many other types of small vesicles, distinguishing each type of vesicle in NSC‐EVs and clarifying their distinct functions might greatly enhance the therapeutic effects of NSC‐EVs in the future. Thirdly, the lack of knowledge on EV content sorting mechanisms significantly limits the development of EV engineering technologies that are able to facilitate selective loading of bioactive molecules with therapeutic effects into NSC‐EVs. Fourthly, targeted strategies and other EV surface modifications are required to promote the accumulation of NSC‐EVs in desired tissues and cells and help them to avoid being phagocytosed by circulating immune cells or being internalized by side organs/tissues. Fifth, utilizing NSCs as a competent cell source for EVs’ mass production is challenging.^197^ Lastly, safe, effective, and high‐yield methodologies for the production and purification of NSC‐EVs need to be established. Probably due to the concerns about safety and lack of essential supporting information, there is currently no clinical trial using NSC‐EVs as shown at clinicaltrials.gov. The pharmacokinetics and pharmacodynamics of NSC‐EVs in humans remain to be elucidated before the clinical application of NSC‐EVs.

Inspiringly, studies that aimed to overcome aforementioned issues have already reported encouraging results. For example, EVs decorated with the CNS‐specific rabies viral glycoprotein (RVG) peptide on their surface by transfecting EV‐generating cells with RVG‐Lamp2b plasmid exhibited excellent CNS targeting capacity through intravenous injection.^198^ Similarly, mannose‐decorated EVs successfully target microglia by binding to mannose receptor (CD206).^199^ PDGFA‐expressing EVs target OPCs by interacting with PDGFRα.^193^ Hence, surface modification techniques successfully confer cell type‐specific targeting capacity to NSC‐EVs, revealing a bright future of NSC‐EV‐based targeted therapy. Additionally, due to ethical/religious issues regarding the application of NSCs, induced NSCs (iNSCs) have been generated to replace NSCs for EV production through direct reprogramming of somatic cells.^137^, ^200^ Comparable effects of NSC‐EVs and iNSC‐derived EVs (iNSC‐EVs) on promoting neuroregeneration, suppressing neuroinflammation, inhibiting neuronal death, and enhancing brain functional recovery were found in an ischemic stroke mouse model, suggesting that iNSC‐EVs are an excellent substitute for NSC‐EVs.^24^

Overall, although there are obstacles that restrict clinical applications of NSC‐EVs, we are hopeful to overcome these challenges with more comprehensive ongoing and planned investigations to make NSC‐EVs a promising therapy for neurological diseases and beyond.

## AUTHOR CONTRIBUTIONS

X.L., X.X., and J.C.Z. conceptualized the manuscript. X.L., Y.Z., X.X., and Y.W. wrote the manuscript. X.L. prepared the figures. All authors read and approved the final manuscript.

## CONFLICT OF INTEREST

The authors declare that they have no known competing financial interests or personal relationships that could have appeared to influence the work reported in this paper.

## ETHICS STATEMENT

Not applicable.

## Data Availability

Not applicable.
